# A Scheme for Ultrasensitive Detection of Molecules with Vibrational Spectroscopy in Combination with Signal Processing

**DOI:** 10.3390/molecules24040776

**Published:** 2019-02-21

**Authors:** Yong Boon Tan, Ian Rongde Tay, Liang Yi Loy, Ke Fun Aw, Zhi Li Ong, Sergei Manzhos

**Affiliations:** 1 Raffles Institution, One Raffles Institution Lane, Singapore 575954, Singapore; tanyongboon1@gmail.com (Y.B.T.); iantayrongde@gmail.com (I.R.T.); 2 NUS High School of Mathematics and Science, 20 Clementi Avenue 1, Singapore 129957, Singapore; h1410074@nushigh.edu.sg (L.Y.L.); h1410011@nushigh.edu.sg (K.F.A.); h1410087@nushigh.edu.sg (Z.L.O.); 3 Department of Mechanical Engineering, National University of Singapore, Block EA #07-08, 9 Engineering Drive 1, Singapore 117576, Singapore

**Keywords:** vibrational spectroscopy, matched filter, signal processing, density functional theory, anharmonicity

## Abstract

We show that combining vibrational spectroscopy with signal processing can result in a scheme for ultrasensitive detection of molecules. We consider the vibrational spectrum as a signal on the energy axis and apply a matched filter on that axis. On the example of a nerve agent molecule, we show that this allows detection of a molecule by its vibrational spectrum, even when the recorded spectrum is completely buried in noise when conventional spectroscopic detection is impossible. Detection is predicted to be possible with signal-to-noise ratios in the recorded spectra as low as 0.1. We have studied the importance of the spectral range used for detection as well as of the quality of the computed spectrum used to program the filter, specifically, the role of anharmonicity, of the exchange correlation functional, and of the basis set. The use of the full spectral range rather than of a narrow spectral window with key vibrations is shown to be advantageous, as well as accounting for anharmonicity.

## 1. Introduction

The detection of molecules is important for civil as well as defense applications. This includes detection of harmful molecules such as chemical warfare (CW) agents (or their precursors or reaction/decay products) or molecules which are indicative of the presence of other controlled substances (explosives, fuels). Such detection is desirable at the lowest possible concentrations, even when such concentrations are not harmful. Extremely low concentrations can be critically harmful, for example, the LC50 dose (the lethal concentration required to kill 50% of the population) of AsF_5_ gas is only 20 ppm; it is desirable to be able to detect harmful molecules at much smaller concentrations than the LC50. While multiple detection techniques exist, they all have their limitations, specifically, in sensitivity and selectivity. For example, detection of molecules by light absorption/photoluminescence properties has been proposed [1] but is very non-selective, as many molecules absorb or luminesce in similar bands. Some methods require a liquid sample, which may not be available; for example, when detecting molecules in the atmosphere or at the border of a territory where release may occur. As stated in a relatively recent review [2], “<available> devices have several limitations, such as low specificity and inability to detect all CW agents. Definitive identification of an agent can be carried out onsite in a mobile analytical laboratory or in an off-site laboratory, and this will generally take many hours. Clinical symptoms and signs in exposed individuals may be the most useful indicators of the likely agent.” The last sentence highlights how important detection is and that available methods are still deficient. One certainly does not want to be in a situation where “clinical symptoms and signs in exposed individuals may be the most useful indicators of the likely agent”.

IR (infrared) detection and other vibrational spectroscopies (for example those which are designed to detect adsorbed molecules such as surface-enhanced Raman [3]) can be very sensitive and selective. Vibrational spectroscopy is a workhorse characterization technique used for species identification in many applications, from atmospheric chemistry to heterogeneous catalysis to Li-ion batteries [4]. It can work in different environments, including ambient air. For example, one can record an IR spectrum by passing an IR laser beam through a relatively extended path in the atmosphere, e.g., by using surveillance aircraft. Vibrational spectroscopy is typically used to monitor a small number of characteristic frequencies of specific functional groups such as -COO and -OH [4,5,6]. However, similar vibrational frequencies can result from the same or other functional groups in very different molecules, especially if molecules interact among themselves or with a substrate [3,4]. This makes assignment unreliable, and multiple other pieces of information and intuition are necessary for a reliable assignment. In addition, a good SNR (signal-to-noise ratio) (certainly >1) of recorded spectra is necessary. That is to say, selectivity and sensitivity are limited.

In the gas phase, when there is little interaction between molecules, one can match the measured spectrum with spectra in the library to detect a molecule. Only in the gas phase does one have the luxury of having a quality reference spectrum. In that case, one can achieve sub-ppm-level sensitivity [7]; it is achieved by using a large number of vibrational transitions (in a wide frequency range) and not only those due to fundamental transitions in key functional groups. The sensitivity drops to a fraction of a wt % in liquid, where the transition frequencies are perturbed/broadened by the environment and where the considered frequency range is typically smaller [8].

Note that one may not have the ability to measure a quality reference spectrum because the chemical can be dangerous or restricted or because the spectrum cannot be measured well (with sufficient resolution, spectral range, and SNR) in the target environment. This is the case for the molecule considered here. In this case, a sufficiently accurate computed spectrum may be used as a reference. Such spectra are computable by using an ab initio approach coupled with a method to solve the vibrational Schrödinger equation. The computed spectra can be perturbed/broadened to mimic environmental effects. For small molecules, various wavefunction-based methods can be used, while for larger molecules, DFT (density functional theory) is often the only practical option [9]. We will use DFT in this article, which is sufficient for the purpose of this work. Vibrational spectra are easily computed in the harmonic approximation; accurate spectra, however, require treatment of anharmonicity and coupling, either perturbatively [10] or by solving the vibrational Schrödinger equation using an analytic potential energy surface representation [11,12] or its discreet samples [13,14,15,16,17]. The perturbative approach is implemented in major ab initio codes and can provide good accuracy in the absence of resonances, it is thus easily applicable for applications and is used here, while methods to properly solve the vibrational Schrödinger equation require more CPU and manpower resources.

In this article, we propose and computationally test the concept of ultrasensitive detection using the vibrational spectrum in a wide frequency range coupled with optimal filtration. The vibrational spectrum of a molecule is not limited to lowest-quanta transitions of a few key functional groups’ models. Considered from the point of view of signal processing, the complete vibrational spectrum (absorption intensity as a function of frequency) even of a small molecule is a very complex signal. For example, while the vibrational spectrum of phosgene (a famous CW agent) is dominated by several strong peaks [18], there are actually 250 transitions just up to 2700 cm^−1^ [15]; many of those transitions are IR inactive, but many are and have a small intensity. The complexity of the entire vibrational spectrum considered as a signal means that it is a unique molecular fingerprint. This signal complexity (and therefore a sharp autocorrelation function) also means that its recovery in the presence of noise (thermal noise in the spectrometer or signals due to external radiation or presence of other species in the environment) could be efficient with signal processing techniques [19] such as matched filtering. This, in turn, means that selectivity and sensitivity could be drastically improved vs existing techniques. To use this idea, one must have accurate benchmarks (reference spectra) of vibrational spectra in a wide excitation energy range to program the matched filter. The reliance of the detector on multiple spectral lines in a wide frequency range means that anharmonicity should be considered when using computed spectra as a reference.

**Figure 1:**
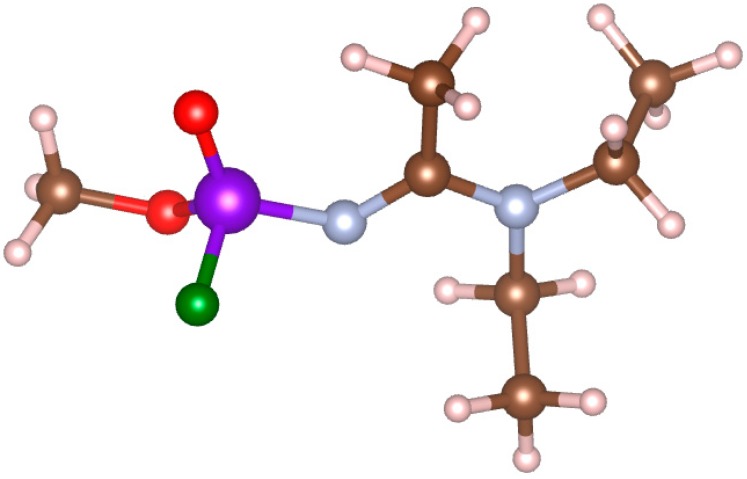
The molecular structure of the A232 nerve agent. Atom color scheme: C, brown; H, pink; N, blue; O, red; P, violet; F, green.

Here, we apply this proposed scheme to detect the vibrational spectrum of the nerve agent A232 (the lay name “Novichok”) in the presence of noise. A232, whose molecular structure is shown in Figure 1, is an organophosphorus compound, which is deadly in tiny concentrations. It inhibits the enzyme acetylcholinesterase and cripples the nervous system [20]. While the molecular structure is known, characterization data are not easily available. We compute the IR spectrum of this molecule with different DFT based approaches, including different functionals, basis sets, and treatment of anharmonicity. We use the highest quality computed spectrum mixed with noise to model the measured spectrum, and spectra computed with all considered levels of theory to program a matched filter in the frequency space. In this way, we study the effect on the detection limit of the quality of the computed spectrum (effect of the functional, the basis, and of the harmonic vs anharmonic approximation) and of the width of the spectrum window used for detection. This approach allows us to completely disambiguate the molecular spectrum from the noise, which would be impossible by e.g., using a measured spectrum, and to include an important limiting case when the computed spectrum exactly models the molecular spectrum present in the input, i.e., to disambiguate the effects on the detection limit due to the use of matched filtering and due to the quality of DFT calculations. In addition, for many dangerous molecules, including the one considered here and generally for molecules which require detection at trace concentrations, high-quality experimental spectra are not available. We find that the molecule can be detected even when noise makes the spectrum visually completely unrecognizable. As expected, we find that lower levels of theory lead to higher detection limits (higher required SNR). We specifically find that anharmonic calculations are much preferred for use as reference spectra, highlighting the practical value of highly accurate computational anharmonic spectroscopy.

## 2. Methods

DFT [21,22] calculations were performed in Gaussian 09 [23] using PBE [24] and B3LYP [25] functionals. Different basis sets were used: 6-31g and 6-311G+(2d,2p). Basis sets of intermediate size were also tried but did not produce new knowledge. Different possible conformers of the A232 molecule were tried and the lowest energy conformer (shown in Figure 1) was used in subsequent calculations. Vibrational spectra were computed in the harmonic and the anharmonic approximations with all (four) combinations of functionals and basis sets. The anharmonic calculations were performed using the 2nd order perturbation theory [10]. The spectra were Gaussian-broadened by 1 cm^−1^ (Gaussian width) for further processing. The calculations were done in a vacuum.

The matched filter was coded in Octave [26]. A matched filter [27] is a linear filter that maximizes the SNR in the presence of a given type of noise. For a discrete signal ${x^{\prime }}_{k}=x^{\prime }\left(k\right)$, where *k* indices discretization points, the output $y_{n}=y\left(n\right)$ is computed as:

(1) $$y_{n}=\sum_{k=-\infty }^{\infty }h\left(n-k\right){x^{\prime }}_{k},$$

where *h* is the filter. The matched filter correlates the received signal (a vector ***x***′ indexed by *k*) with a filter ***h*** (another vector) that is parallel with the signal, maximizing the inner product. This is achieved when $h=\alpha R_{\xi }^{-1}x$, where $R_{\xi }$ is the covariance of the noise and α a normalization constant. The vector ***x*** is the useful (expected) signal component of the input ***x***′, which is deteriorated by the noise ξ: $x^{\prime }=x+\xi$. For white noise assumed here, we can put h=x and matched filtering becomes equivalent to correlating the received signal with the expected signal with the output ***y*** proportional to the signal’s autocorrelation function. The technique is, therefore, the more powerful the more complex the shape of ***x*** is i.e., the sharper its autocorrelation function, ideally approaching the delta function for very complex-shaped signals. The quality of the detection deteriorates if there is a mismatch between ***h*** and ***x***. A popular example is relatively easy radar detection of stealth aircraft using complex-shaped, broadband signals. In signal processing applications, *k* usually indexes time intervals.

In this work, we consider the vibrational spectrum as such a complex signal with a sharp autocorrelation function when considered along the frequency axis. We, therefore, apply the matched filter along the frequency axis. The highest quality computed spectrum (that with B3LYP functional and 6-311G+(2d,2p) basis set and with the anharmonic corrections) is used to emulate the signal ***x***; it plays the role of a benchmark. It is mixed with white noise and is detected using a spectrum computed with different levels of theory as ***h***. We also test performing the detection in the entire relevant spectral range (set here from 0 to 4000 cm^−1^) vs. a frequency window (from 680 to 2000 cm^−1^ where the fundamentals corresponding to vibrations of most bonds and angles lie).

The frequency axis was discretized with a step of 0.061 cm^−1^ using 2^16^ points (*k* values) up to 4000 cm^−1^. Under spectral broadening of 1 cm^−1^ used here, this provided converged results (no changes were detected with a finer discretization). Note that 0.061 cm^−1^ is a digitization resolution and the 1 cm^−1^ broadening models the spectrum resolution. Both are easily achievable in measurements and data processing. The white noise was subjected to the same broadening. Unsigned noise was added to the spectrum for computational simplicity. The peak in the autocorrelation function was detected by looking for any elements of ***y*** which exceed *N* × *σ* where *σ* is the standard deviation of ***y*** and *N* is a chosen parameter. The detection limit is defined as a noise level with which the rate of false negatives reaches 50%. False positives, in this case, are due to a finite probability of satisfying the criterion $y_{k}>N\times \sigma$ with the noise in the absence of the signal ***x***. To reduce the rate of false positives, the peak search was performed in the window of width 300 and 80 cm^−1^ (when using the full spectrum range and the window 680–2000 cm^−1^, respectively) around the mid-point of ***y***. When ***h*** = ***x*** (i.e., when the same level of theory is used for the signal and for the filter), the peak was exactly at the mid-point. When different levels of theory are used, the peak may be off-center; the peak detection window was chosen for the worst mismatch among all cases. The rate of false positives was computed by using noise-only inputs. With each instance of noise (for both signal-containing and noise-only inputs), 50 numeric experiments were performed. The corresponding SNR is defined as the ratio of the root mean square of the signal ***x*** (the spectrum) and the standard deviation of the noise, both under the same broadening of 1 cm^−1^. With 50 experiments, the statistical spread of SNR estimates were within 0.1, which is sufficient for our purposes.

## 3. Results and Discussion

**Figure 2:**
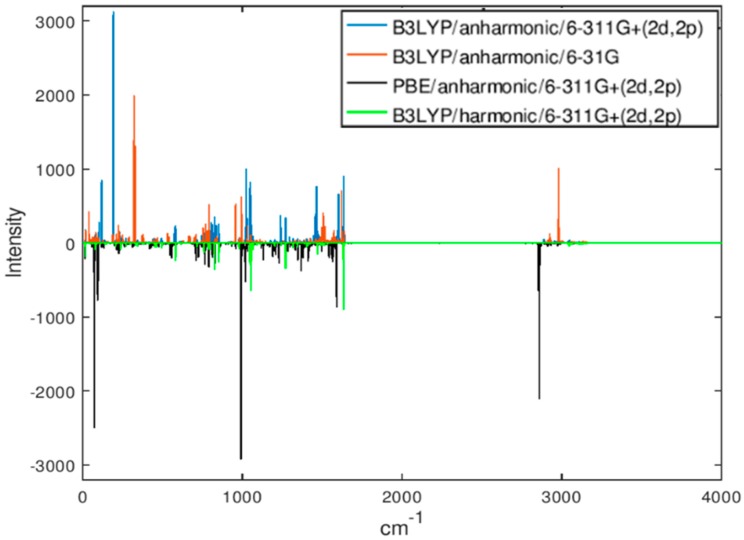
IR spectra of the A232 molecule computed with different levels of theory. Spectra are Gaussian broadened by 1 cm^−1^ (Gaussian width). Some spectra are plotted in the negative for better visibility. The intensities are in 10^−40^ esu^2^ cm^2^.

**Figure 3:**
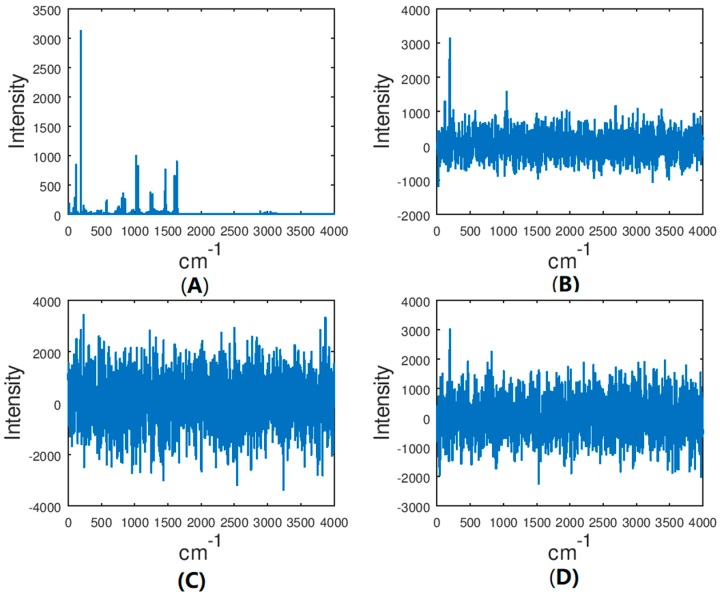
The IR spectrum of A232 computed at the B3LYP/anharmonic/6-311G+(2d,2p) level and summed with the white noise of amplitudes (clockwise from top left) (**A**) 0, (**B**) 500, (**C**) 1500, and (**D**) 1000.

**Figure 4:**
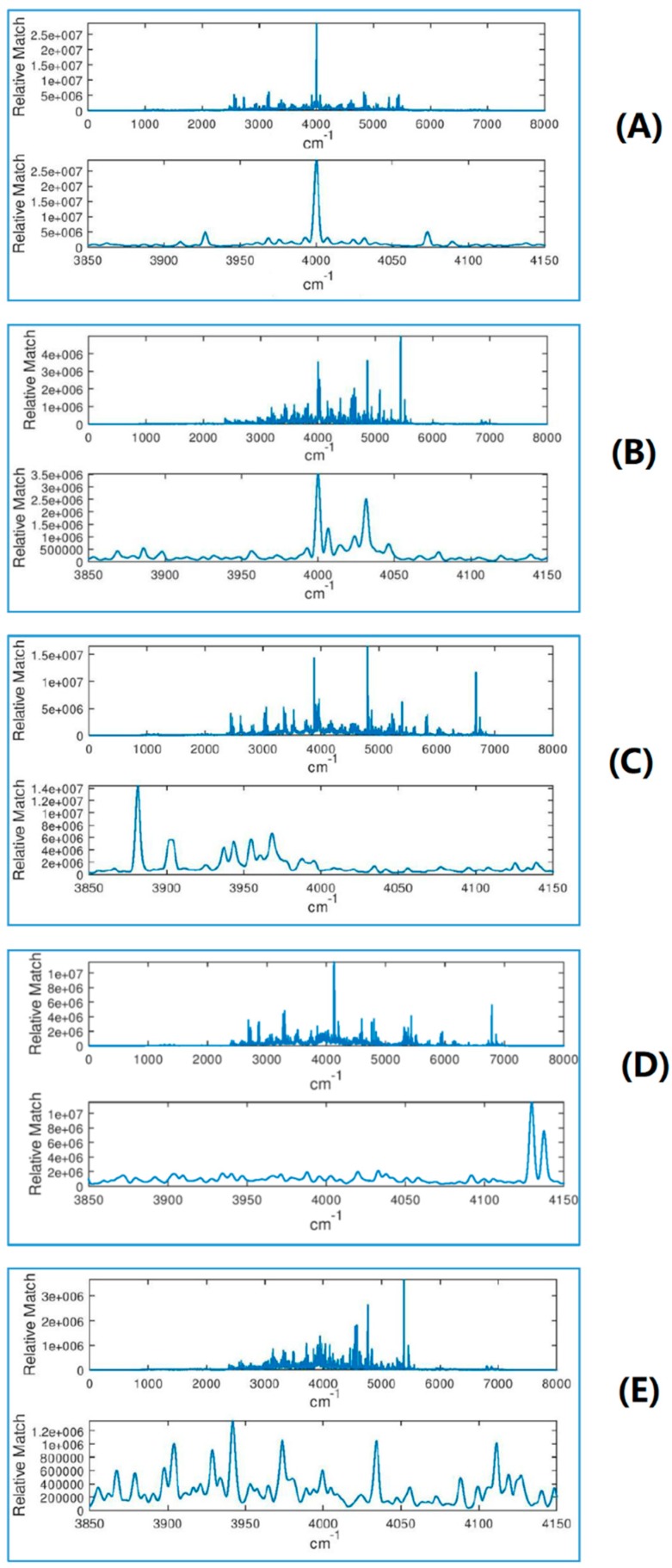
The correlation function of the reference spectrum (anharmonic spectrum computed with B3LYP/6-311G+(2d,2p)) with spectra computed at different levels of theory, using the full spectral range 0–4000 cm^−1^. From top to bottom: (**A**) B3LYP/anharmonic/6-311G+(2d,2p) (i.e., autocorrelation), (**B**)_B3LYP/harm/6-311G+(2d,2p), (**C**) PBE/anharmonic/6-311G+(2d,2p), (**D**) B3LYP/anharmonic/6-31G, and (**E**) PBE/harmonic/6-31G. The top panel in each pair of plots is for the entire signal ***y*** and the bottom panel is zoomed around the center, where a peak is expected.

Molecular geometry computed at the B3LYP/6-311G+(2d,2p) level of theory as well as vibrational spectra computed with all combinations of functional and basis sets are given in the Supporting Information. Figure 2 shows computed IR spectra for several combinations of basis and functional, harmonic and anharmonic. Figure 3 shows the (benchmark) spectrum computed at the highest level of theory (B3LYP/anharmonic/6-311G+(2d,2p)) with different levels of noise. (The units of intensity, which are 10^−40^ esu^2^ cm^2^, are dropped in the subsequent discussion as they are unimportant and make no difference to the conclusions as long as all calculations are done at the same scale and with the same broadening, which is the case). At noise levels on the order of 500, the spectrum is completely unusable for assignment. This case corresponds to a value of SNR as defined above of about 0.25. Examples of correlation functions (filter output ***y***) of the benchmark spectrum with spectra computed at different levels of theory are shown in Figure 4. The top pair of plots show the autocorrelation function which possesses a sharp peak in the middle, which is due to the complex shape of ***x***. The delta function–like nature of the autocorrelation function of a vibrational spectrum of a polyatomic molecule makes it well suited for applying matched filtering and therefore can permit detection in the presence of high levels of noise. The following three pairs of panels show the effect of errors in the computed spectrum (which programs the filter ***h***) on the quality of the peak in ***y*** due to the use of the harmonic approximation, of a less accurate exchange-correlation functional, and of a small basis set, respectively. The final pair of plots (at the PBE/harmonic/6-31G level) show the combined effect of these sources of error. Note that the application of Equation 1 results in the range of the abscissa values for ***y*** which is double that of the original spectrum, i.e., 8000 cm^−1^, with the autocorrelation peak centered at 4000 cm^−1^.

As expected, lower levels of theory deteriorate the quality of the output of the filter (height and position of the main peak used for detection). The effects of various components of the error are different. For example, use of the harmonic approximation (other approximations being equal) reduces the peak height by about an order of magnitude but does not shift significantly its position. The use of the PBE functional (other parameters being equal) reduces the peak height “only” by a factor of 2 but shifts its position by more than 100 cm^−1^ with respect to the position to the autocorrelation function’s peak. Qualitatively, a similar effect is observed when using a smaller basis (other parameters being equal). All lower-level approximations lead to the appearance of strong off-center peaks. The combined effect of all these sources of error (bottom pair of plots) is a peak height reduced by about a factor of 20 and shifted by more than 100 cm^−1^, with multiple satellite peaks, some exceeding in height the central peak. Overall, the results shown in Figure 4 suggest that anharmonicity treatment is important as well as proper choices of functional and basis set, and that one must allow for a “window” on the order of ±200 cm^−1^ for peak detection.

**Figure 5:**
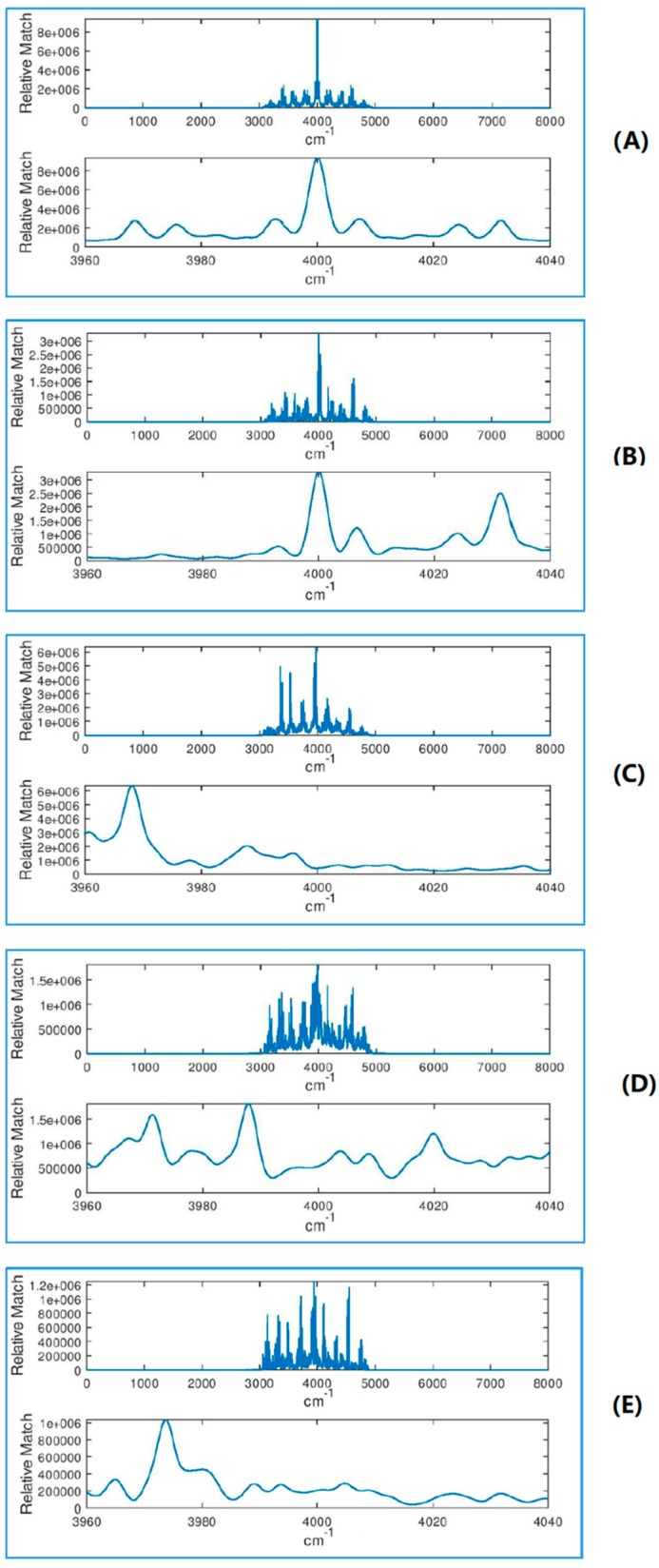
The correlation function of the reference spectrum (anharmonic spectrum computed with B3LYP/6-311G+(2d,2p)) with spectra computed at different levels of theory within a detection window of 680–2000 cm^−1^. From top to bottom: (**A**) B3LYP/anharm/6-311G+(2d,2p) (i.e., autocorrelation), (**B**) B3LYP/harm/6-311G+(2d,2p), (**C**) PBE/anharm/6-311G+(2d,2p), (**D**) B3LYP/anharm/6-31G, and (**E**) PBE/harm/6-31G. The top panel in each pair of plots is for the entire signal ***y*** and the bottom panel is zoomed around the center, where a peak is expected.

We also explored detection in a selected spectral window of 680–2000 cm^−1^. In this case, components of ***x***′ and of ***h*** outside this range were set to zero. The resulting correlation functions (filter output ***y***) of the benchmark spectrum with spectra computed at different levels of theory are shown in Figure 5. The use of a spectral window reduced the peak height in the autocorrelation function by about a factor of two but it also reduced the degree of relative deterioration of the height and position of the main peak with approximate computational schemes. For example, the use of the harmonic approximation (other parameters being equal) reduced the peak height by about a factor of 2½. The use of the PBE functional (other parameters being equal) caused the peak’s shift but hardly reduced its height. The use of a small basis (other parameters being equal), however, reduced the peak height by about a factor of four and had a worse effect than in the case of a full spectral range. The combined effect of all these sources of error is a peak weakened by an order of magnitude with multiple and strong satellite peaks. It may appear, therefore, based on Figure 5 that using a limited spectral range may be beneficial if the filter is programmed with a computed spectrum using a sufficiently complete basis set.

**Figure 6:**
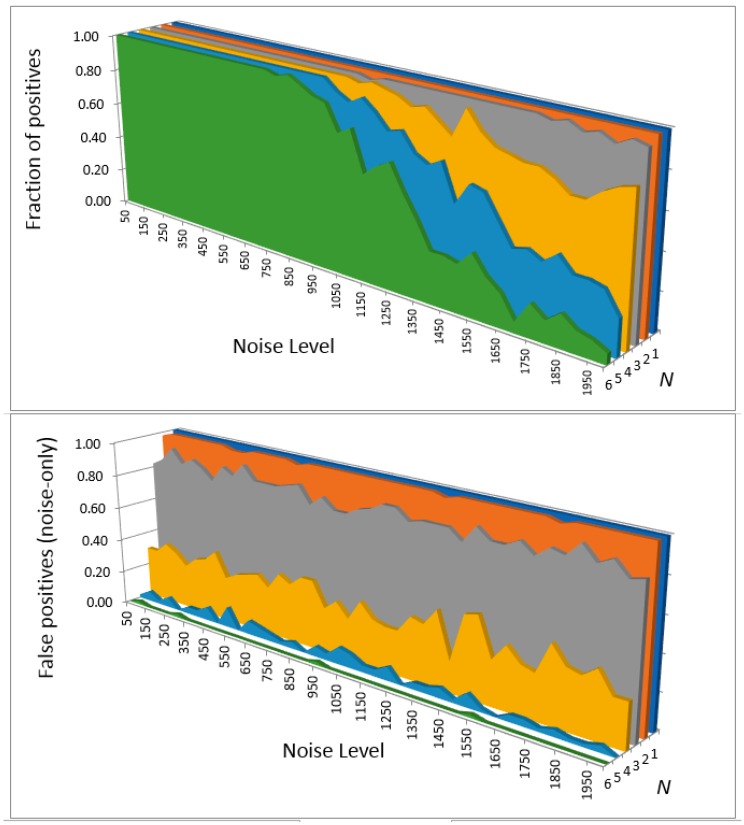
The fraction of positive detection outcomes (detection criterion *y* > *N* × *σ* satisfied) for different levels of noise and *N*, for detection in the full spectral range of 0–4000 cm^−1^. The top panel is for the inputs consisting of the molecular spectrum and noise and the bottom panel for noise-only inputs. The filter is programmed with a spectrum computed at the B3LYP/anharm/6-311G+(2d,2p) level.

**Figure 7:**
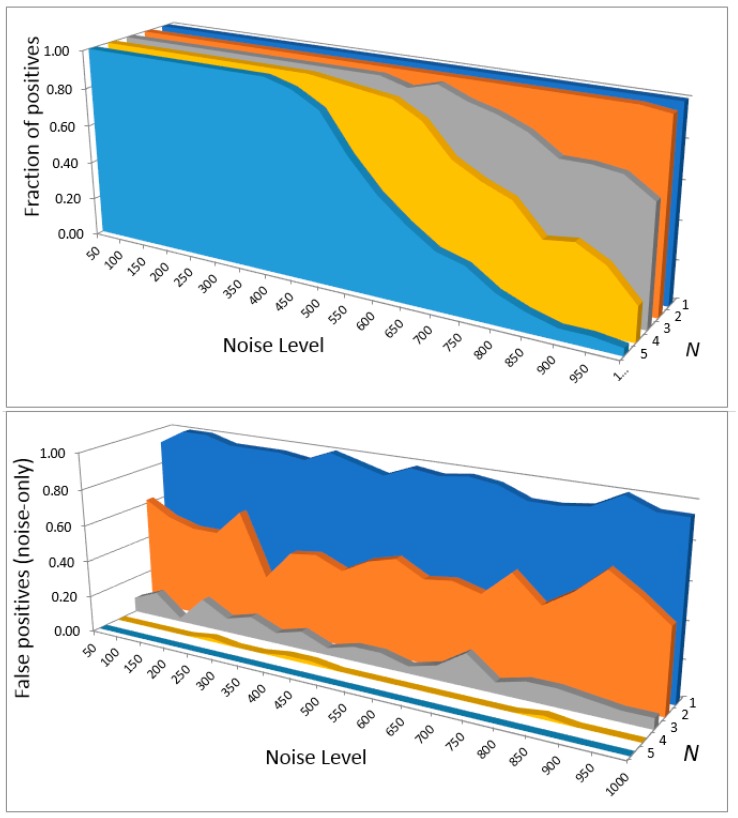
The fraction of positive detection outcomes (detection criterion *y* > *N* × *σ* satisfied) for different levels of noise and *N*, for detection in the full spectral window 680–2000 cm^−1^. The top panel is for the inputs consisting of the molecular spectrum and noise and the bottom panel for noise-only inputs. The filter is programmed with a spectrum computed at the B3LYP/anharm/6-311G+(2d,2p) level.

We computed the detection limits and corresponding SNR ratios when detecting the benchmark spectrum deteriorated with different levels of noise with a filter programmed with a spectrum computed at different levels of theory, using the full spectrum up to 4000 cm^−1^ or a detection window of 680–2000 cm^−1^. The detection thresholds were determined by comparing the rate of positives with the rate of false positives (on noise-only inputs) and correspond to 50% probability of detection. Different *N* were tried in the peak detection criterion $y_{k}>N\times \sigma$. This criterion was applied in the window of ±150 (when using full spectral range) or ±40 cm^−1^ (when using a window 680–2000 cm^−1^), based on the results of Figure 4 and Figure 5. Examples of these calculations when programming ***h*** with the spectrum computed at the B3LYP/anharmonic/6-311G+(2d,2p) level are shown in Figure 6 for the case of full spectrum detection and in Figure 7 for detection in the spectral window.

The results for all cases are shown in Table 1. The results show that with the criterion *y* > 6*σ*, one can achieve a sufficiently low rate of false positives of <<0.1. When the filter response function is programmed with a high quality computed spectrum, the detection threshold SNR can then be as low as about 0.1, corresponding the “noisiest” panel of Figure 3 which appears to consist entirely of noise. This is achieved by using the entire spectral range considered here (0–4000 cm^−1^). Detection in a narrower window, in spite of some advantages listed above, results in higher required SNR, albeit reliable detection should still be possible with SNR < 0.5. This highlights the fact that it is really in a wide spectral range that the molecular vibrational spectrum has unique, fingerprint-like quality.

The broadening of 1 cm^−1^, which we used for the systematic tests described above was chosen to balance resolutions typically achieved in the gas phase (sub-cm^−1^) and at surfaces and in other aggregate states (typically 1–4 cm^−1^) [3,4,5]. We conducted selective calculations with other broadening values to confirm that the conclusions are not skewed by the choice of a specific broadening value. For example, when using a broadening of 2 cm^−1^, the detection limit when using a spectrum computed at the B3LYP/harmonic/6-311G+(2d,2p) level to program the filter is, in terms of SNR (using full spectral range): 0.32 when using a 5*σ* criterion and 0.39 using a 6*σ* criterion, i.e., somewhat higher as expected due to less sharp features of the spectrum and of the autocorrelation function with larger broadening (lower spectral resolution). We also conducted a test of the effect of the presence of another species in the environment on the detection capability of the filter: we mixed in the input the vibrational spectrum of a water molecule. For consistency, the H_2_O spectrum was computed at the same level of theory as the reference A232 input spectrum and broadened the same way (by 1 cm^−1^) as the input spectrum and the noise. This test ignored ro-vibrational contributions which are important in the case of a light molecule like water, it is therefore indicative of the effect of the presence of another species without being quantitative. The intensities in the water spectrum were multiplied by 10 to reflect the fact that concentrations of ambient species may be much higher than that of the target molecule. The detection limit when using a spectrum computed at the B3LYP/harmonic/6-311G+(2d,2p) level to program the filter is, in terms of SNR (using full spectral range): 0.23 when using a 5*σ* criterion and 0.28 using a 6*σ* criterion, i.e., on the same order as that without water. In general, it is expected that the selectivity should remain high in the presence of other well-structured signals in the input such as well-resolved spectra of other molecules, although of course in applications, specific studies should be conducted for specific environments where particular species are present.

## 4. Conclusions

We explored computationally a scheme for detection of molecules that combines vibrational spectroscopy with optimal filtration. We considered the vibrational spectrum of a molecule in a wide spectral range as its unique fingerprint. The spectral shape presents a complex signal with a sharp autocorrelation function, which makes it suitable for detection using matched filtering. We showed that this scheme can be applied for detection of molecules at low concentrations where the signal-to-noise ratio of recorded spectra is very low. Specifically, we considered detection of a nerve agent A232 which is lethally harmful at trace concentrations and showed that the proposed scheme could detect the presence of the molecule when the SNR is as low as 0.1 i.e., when the recorded spectrum is completely buried in noise. A232 is also an example of a molecule where experimental reference spectra needed to code the matched filter are not accessible; this is true for most dangerous substances. The matched filter can be programmed by a computed spectrum. We studied the sensitivity of the scheme when different levels of theory are used to compute the spectrum for the matched filter such as different exchange-correlation functionals, basis sets, and treatment of anharmonicity. The sensitivity of the detector is, as expected, improved with higher levels of theory. Specifically, anharmonic calculations are shown to be much preferred; this highlights the practical value of computational anharmonic spectroscopy and suggests that methods properly solving the vibrational Schrödinger equation (as opposed to perturbative treatment used here) on the extended potential energy surface (either analytic or discretely sampled with ab initio calculations) should be explored for this application. In general, this work highlights the value of highly accurate computational vibrational spectroscopy. We also observed the advantage of considering a wide spectral range: detection in a narrower spectral window, in spite of some advantages, results in higher required SNR, albeit reliable detection should still be possible with SNR < 0.5. This highlights the fact that it is really in a wide spectral range that the molecular vibrational spectrum has unique, fingerprint-like quality.

In this work, we have presented an idea that could become further developed and validated. We used moderate accuracy DFT based setups for a proof of principle. For sufficiently small molecules, wavefunction methods could be used. We also ignored rotational contributions to the spectra. For relatively large molecules like the one considered here, it may not be practical to treat rotational lines individually, and appropriate broadening could be used instead. For sufficiently small molecules where rotational resolution is possible, the ro-vibrational spectrum can offer increased complexity and more delta function-like autocorrelation function, which could lead to even more sensitive schemes. It will also be important to properly treat rotational contributions for a quantitative assessment of the effects of the presence of small molecules in the environment. Our preliminary tests showed that a larger broadening leads to a larger detection threshold, as is expected with a less delta function—line autocorrelation function in this case. The use of the method may, therefore, be less advantageous in liquid and solid state but deserves to be explored there.

## Figures and Tables

**Table 1:** Detection levels (noise levels and corresponding SNR values in parentheses) when using spectra computed at different levels of theory to program the filter. The reference spectrum computed at the highest level of theory is used to model the signal deteriorated by noise. Lower level approximations vs. the reference spectrum are highlighted in bold. The results are shown for the most promising peak detection criteria (*N* × *σ*).

Method	Detection Window		Full Spectrum	
	*N* × *σ* Detection Criteria			
	4*σ*	5*σ*	5*σ*	6*σ*
	Detection Threshold (SNR)			
B3LYP/anharmonic/6-311G+(2d,2p)	819 (0.17)	608 (0.22)	1600 (0.08)	1317 (0.10)
B3LYP/**harmonic**/6-311G+(2d,2p)	533 (0.25)	388 (0.34)	684 (0.20)	465 (0.29)
B3LYP/anharmonic/**6-31G**	250 (0.56)	143 (0.97)	869 (0.16)	672 (0.20)
**PBE**/anharmonic/6-311G+(2d,2p)	373 (0.37)	304 (0.46)	700 (0.20)	522 (0.26)
**PBE/harmonic/6-31G**	190 (0.72)	156 (0.85)	357 (0.38)	221 (0.62)

## Supplementary Materials

Supplementary Materials are available online. Molecular spectra computed with different levels of theory (a .xlsx file) and molecular structure of A232 (a .xyz file).
